# Supplementary material for: Drug-Induced Immune Thrombocytopenia Toxicity Prediction Based on Machine Learning
Source: Pharmaceutics. 2022 Apr 26;14(5):943. doi: 10.3390/pharmaceutics14050943 (PMC9143325; doi:10.3390/pharmaceutics14050943)
Supplement: Supplementary file 1 [file pharmaceutics-14-00943-s001.zip › pharmaceutics-1682247-supplementary.pdf]

Drug-induced immune thrombocytopenia toxicity prediction based on machine learning

SUPPLEMENTARY INFORMATION

Binyou Wang [1, †], Xiaoqiu Tan [2,3,4,†],Jianmin Guo [2], Ting Xiao [2], Yan Jiao [5], Junlin Zhao [1], Jianming Wu [6,7,], Yiwei Wang [2,3,4,*]*

[1]School of Pharmacy, Southwest Medical University, Luzhou, 646000, China.

[2]School of Basic Medical Science, Southwest Medical University, Luzhou, 646000, China.

[3]Key Laboratory of Medical Electrophysiology, Southwest Medical University, Luzhou 646000, China.

[4]Ministry of Education & Medical Electrophysiological Key Laboratory of Sichuan Province, Institute of Cardiovascular Research, Southwest Medical University, Luzhou 646000, China.

[5]State Key Laboratory of Biotherapy and Cancer Center, West China Hospital, Sichuan University, Chengdu 610041, China.

[6]Sichuan Key Medical Laboratory of New Drug Discovery and Druggability Evaluation; School of Pharmacy, Southwest Medical University, Luzhou,646000 China.

[7]Luzhou Key Laboratory of Activity Screening and Druggability Evaluation for Chinese Materia Medica, School of Pharmacy, Southwest Medical University, Luzhou,646000 China.

† These authors contributed equally to this work.

* Corresponding authors: Yiwei Wang, Jianming Wu

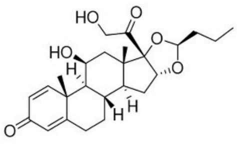

FP1: Budesonide

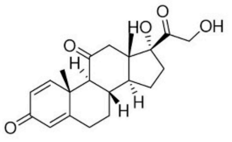

FP2: Prednisone

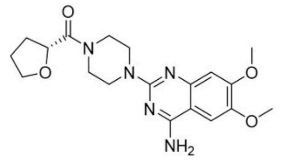

FP3: Terazosin

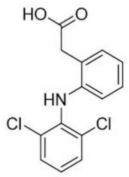

FP4: Diclofenac

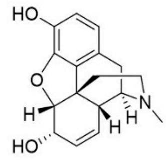

FP5: Morphine

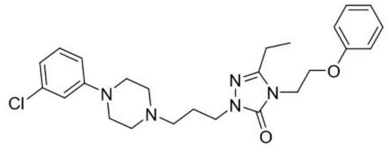

FP6: Nefazodone

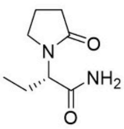

FP7: Levetiracetam

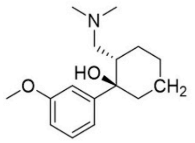

FP8: Tramadol

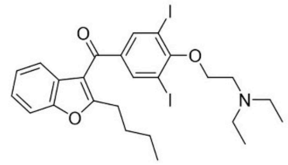

FN1: Amiodarone

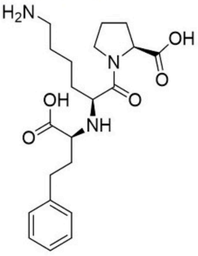

FN2: Lisinopril

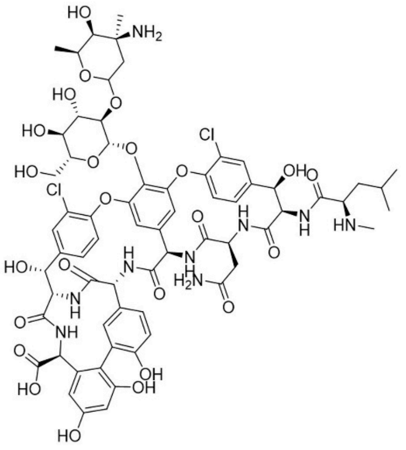

FN3: Vancomycin

Figure S1. Misclassified compounds of the RDMD+PubChem-k-NN model on the external validation set.

Table S1. Details of the training set and external validation set compounds.

**Training set**		
**Name**	**Class**	**SMILES**
ACETAMINOPHEN	1	CC(=O)NC1=CC=C(O)C=C1
ACYCLOVIR	0	NC1=NC(=O)C2=C(N1)N(COCCO)C=N2
ALBUTEROL	0	CC(C)(C)NCC(O)C1=CC(CO)=C(O)C=C1
ALENDRONATE	0	NCCCC(O)(P(O)(O)=O)P(O)(O)=O
ALLOPURINOL	0	OC1=NC=NC2=C1C=NN2
ALPRAZOLAM	0	CC1=NN=C2CN=C(C3=CC=CC=C3)C3=C(C=CC(Cl)=C3)N12
AMITRIPTYLINE	1	CN(C)CCC=C1C2=CC=CC=C2CCC2=CC=CC=C12
AMLODIPINE	0	CCOC(=O)C1=C(COCCN)NC(C)=C(C1C1=CC=CC=C1Cl)C(=O)OC
AMOXICILLIN	1	[H][C@]12SC(C)(C)[C@@H](N1C(=O)[C@H]2NC(=O)[C@H](N)C1=CC=C(O)C=C1)C(O)=O
AMPHOTERICIN B	0	[H][C@]12C[C@@H](O[C@@H]3O[C@H](C)[C@@H](O)[C@H](N)[C@@H]3O)\C=C\C=C\C=C\C=C\C=C\C=C\C=C\[C@H](C)[C@@H](O)[C@@H](C)[C@H](C)OC(=O)C[C@H](O)C[C@H](O)CC[C@@H](O)[C@H](O)C[C@H](O)C[C@](O)(C[C@H](O)[C@H]1C(O)=O)O2
AMPICILLIN	1	[H][C@]12SC(C)(C)[C@@H](N1C(=O)[C@H]2NC(=O)[C@H](N)C1=CC=CC=C1)C(O)=O
ARGATROBAN	1	C[C@@H]1CCN([C@H](C1)C(O)=O)C(=O)[C@H](CCCNC(N)=N)NS(=O)(=O)C1=CC=CC2=C1NCC(C)C2
ASPIRIN	1	CC(=O)OC1=CC=CC=C1C(O)=O
ATORVASTATIN	0	CC(C)C1=C(C(=O)NC2=CC=CC=C2)C(=C(N1CC[C@@H](O)C[C@@H](O)CC(O)=O)C1=CC=C(F)C=C1)C1=CC=CC=C1
AZACITIDINE	1	NC1=NC(=O)N(C=N1)[C@@H]1O[C@H](CO)[C@@H](O)[C@H]1O
BENZTROPINE	0	[H][C@]12CC[C@]([H])(C[C@@]([H])(C1)OC(C1=CC=CC=C1)C1=CC=CC=C1)N2C
BIVALIRUDIN	0	CC[C@H](C)[C@H](NC(=O)[C@H](CCC(O)=O)NC(=O)[C@H](CCC(O)=O)NC(=O)[C@H](CC1=CC=CC=C1)NC(=O)[C@H](CC(O)=O)NC(=O)CNC(=O)[C@H](CC(N)=O)NC(=O)CNC(=O)CNC(=O)CNC(=O)CNC(=O)[C@@H]1CCCN1C(=O)[C@H](CCCNC(N)=N)NC(=O)[C@@H]1CCCN1C(=O)[C@H](N)CC1=CC=CC=C1)C(=O)N1CCC[C@H]1C(=O)N[C@@H](CCC(O)=O)C(=O)N[C@@H](CCC(O)=O)C(=O)N[C@@H](CC1=CC=C(O)C=C1)C(=O)N[C@@H](CC(C)C)C(O)=O

BUPROPION	1	CC(NC(C)(C)C)C(=O)C1=CC(Cl)=CC=C1
BUSPIRONE	0	O=C1CC2(CCCC2)CC(=O)N1CCCCN1CCN(CC1)C1=NC=CC=N1
CAPTOPRIL	0	C[C@H](CS)C(=O)N1CCC[C@H]1C(O)=O
CARBAMAZEPINE	1	NC(=O)N1C2=CC=CC=C2C=CC2=CC=CC=C12
CARBIDOPA	0	C[C@@](CC1=CC(O)=C(O)C=C1)(NN)C(O)=O
CARVEDILOL	0	COC1=CC=CC=C1OCCNCC(O)COC1=CC=CC2=C1C1=CC=CC=C1N2
CASPOFUNGIN	0	[H][C@@]12C[C@@H](O)CN1C(=O)[C@@H](NC(=O)[C@]([H])(C[C@@H](O)[C@@H](NCCN)NC(=O)[C@@H]1[C@@H](O)CCN1C(=O)[C@@H](NC(=O)[C@@H](NC2=O)[C@H](O)[C@@H](O)C1=CC=C(O)C=C1)[C@H](O)CCN)NC(=O)CCCCCCCC[C@@H](C)C[C@@H](C)CC)[C@@H](C)O
CEFADROXIL	1	[H][C@]12SCC(C)=C(N1C(=O)[C@H]2NC(=O)[C@H](N)C1=CC=C(O)C=C1)C(O)=O
CEFEPIME	1	C[N+]1(CCCC1)CC2=C(N3[C@@H]([C@@H](C3=O)NC(=O)/C(=N\OC)/C4=CSC(=N4)N)SC2)C(=O)[O-]
CEFPODOXIME	1	[H][C@]12SCC(COC)=C(N1C(=O)[C@H]2NC(=O)C(=N/OC)\C1=CSC(N)=N1)C(O)=O
CEFTIZOXIME	1	CO/N=C(\C(=O)N[C@@H]1C(=O)N2C(C(=O)O)=C(CSc3nc(=O)c(O)nn3C)CS[C@H]12)c1csc(N)n1
CEFTRIAXONE	1	[H][C@]12SCC(CSC3=NC(=O)C(=O)NN3C)=C(N1C(=O)[C@H]2NC(=O)C(=N/OC)\C1=CSC(N)=N1)C(O)=O
CEFUROXIME	0	[H][C@]12SCC(COC(N)=O)=C(N1C(=O)[C@H]2NC(=O)C(=N/OC)\C1=CC=CO1)C(O)=O
CELECOXIB	1	CC1=CC=C(C=C1)C1=CC(=NN1C1=CC=C(C=C1)S(N)(=O)=O)C(F)(F)F
CEPHALEXIN	1	[H][C@]12SCC(C)=C(N1C(=O)[C@H]2NC(=O)[C@H](N)C1=CC=CC=C1)C(O)=O
CHLORPROMAZINE	0	CN(C)CCCN1C2=CC=CC=C2SC2=C1C=C(Cl)C=C2
CIMETIDINE	0	CN\C(NCCSCC1=C(C)NC=N1)=N\C#N
CIPROFLOXACIN	1	OC(=O)C1=CN(C2CC2)C2=CC(N3CCNCC3)=C(F)C=C2C1=O
CITALOPRAM	0	CN(C)CCCC1(OCC2=C1C=CC(=C2)C#N)C1=CC=C(F)C=C1
CLARITHROMYCIN	0	[H][C@@]1(C[C@@](C)(OC)[C@@H](O)[C@H](C)O1)O[C@H]1[C@H](C)[C@@H](O[C@]2([H])O[C@H](C)C[C@@H]([C@H]2O)N(C)C)[C@@](C)(C[C@@H](C)C(=O)[C@H](C)[C@@H](O)[C@](C)(O)[C@@H](CC)OC(=O)[C@@H]1C)OC
CLAVULANATE POTASSIUM	0	O=C([C@@H](/C(O[C@]1([H])C2)=C/CO)N1C2=O)O[K]

CLINDAMYCIN	1	[H][C@@](NC(=O)[C@@H]1C[C@@H](CCC)CN1C)([C@H](C)Cl)[C@@]1([H])O[C@H](SC)[C@H](O)[C@@H](O)[C@H]1O
CLONIDINE	0	ClC1=CC=CC(Cl)=C1NC1=NCCN1
CLOPIDOGREL	0	[H][C@@](N1CCC2=C(C1)C=CS2)(C(=O)OC)C1=CC=CC=C1Cl
COLCHICINE	0	COC1=CC2=C(C(OC)=C1OC)C1=CC=C(OC)C(=O)C=C1C(CC2)NC(C)=O
CYCLOSPORINE	1	CC[C@@H]1NC(=O)[C@H]([C@H](O)[C@H](C)C\C=C\C)N(C)C(=O)[C@H](C(C)C)N(C)C(=O)[C@H](CC(C)C)N(C)C(=O)[C@H](CC(C)C)N(C)C(=O)[C@@H](C)NC(=O)[C@H](C)NC(=O)[C@H](CC(C)C)N(C)C(=O)[C@@H](NC(=O)[C@H](CC(C)C)N(C)C(=O)CN(C)C1=O)C(C)C
DAPSONE	0	NC1=CC=C(C=C1)S(=O)(=O)C1=CC=C(N)C=C1
DAPTOMYCIN	1	CCCCCCCCCC(=O)N[C@@H](CC1=CNC2=C1C=CC=C2)C(=O)N[C@H](CC(N)=O)C(=O)N[C@@H](CC(O)=O)C(=O)N[C@H]1[C@@H](C)OC(=O)[C@H](CC(=O)C2=CC=CC=C2N)NC(=O)[C@@H](NC(=O)[C@@H](CO)NC(=O)CNC(=O)[C@H](CC(O)=O)NC(=O)[C@@H](C)NC(=O)[C@H](CC(O)=O)NC(=O)[C@H](CCCN)NC(=O)CNC1=O)[C@H](C)CC(O)=O
DEXAMETHASONE	1	[H][C@@]12C[C@@H](C)[C@](O)(C(=O)CO)[C@@]1(C)C[C@H](O)[C@@]1(F)[C@@]2([H])CCC2=CC(=O)C=C[C@]12C
DIAZEPAM	0	CN1C2=C(C=C(Cl)C=C2)C(=NCC1=O)C1=CC=CC=C1
DIGOXIN	0	[H][C@]12CC[C@]3([H])[C@]([H])(C[C@@H](O)[C@]4(C)[C@H](CC[C@]34O)C3=CC(=O)OC3)[C@@]1(C)CC[C@@H](C2)O[C@H]1C[C@H](O)[C@H](O[C@H]2C[C@H](O)[C@H](O[C@H]3C[C@H](O)[C@H](O)[C@@H](C)O3)[C@@H](C)O2)[C@@H](C)O1
DILTIAZEM	0	COC1=CC=C(C=C1)[C@@H]1SC2=C(C=CC=C2)N(CCN(C)C)C(=O)[C@@H]1OC(C)=O
DIPHENHYDRAMINE	1	CN(C)CCOC(C1=CC=CC=C1)C1=CC=CC=C1
DOBUTAMINE	1	CC(CCC1=CC=C(O)C=C1)NCCC1=CC(O)=C(O)C=C1
DOCUSATE	0	CCCCC(CC)COC(=O)CC(C(=O)OCC(CC)CCCC)S(O)(=O)=O
DONEPEZIL	0	COC1=C(OC)C=C2C(=O)C(CC3CCN(CC4=CC=CC=C4)CC3)CC2=C1
DOPAMINE	0	NCCC1=CC(O)=C(O)C=C1
DOXAZOSIN	0	COC1=C(OC)C=C2C(N)=NC(=NC2=C1)N1CCN(CC1)C(=O)C1COC2=CC=CC=C2O1
DOXYCYCLINE	1	[H][C@@]12[C@@H](C)C3=CC=CC(O)=C3C(=O)C1=C(O)[C@]1(O)C(=O)C(C(N)=O)=C(O)[C@@H](N(C)C)[C@]1([H])[C@H]2O
ENALAPRIL	0	CCOC(=O)[C@H](CCC1=CC=CC=C1)N[C@@H](C)C(=O)N1CCC[C@H]1C(O)=O
ESCITALOPRAM	0	CN(C)CCC[C@]1(OCC2=C1C=CC(=C2)C#N)C1=CC=C(F)C=C1

ESOMEPRAZOLE	1	COC1=CC2=C(NC(=N2)[S@@](=O)CC2=NC=C(C)C(OC)=C2C)C=C1
ETHAMBUTOL	1	CC[C@@H](CO)NCCN[C@@H](CC)CO
ETHOXISUXIMIDE	1	O=C(C(C)(CC)C1)NC1=O
EZETIMIBE	0	[H][C@]1(CC[C@H](O)C2=CC=C(F)C=C2)C(=O)N(C2=CC=C(F)C=C2)[C@]1([H])C1=CC=C(O)C=C1
FAMOTIDINE	0	NC(N)=NC1=NC(CSCCC(N)=NS(N)(=O)=O)=CS1
FELBAMATE	1	NC(=O)OCC(COC(N)=O)C1=CC=CC=C1
FELODIPINE	1	CCOC(=O)C1=C(C)NC(C)=C(C1C1=C(Cl)C(Cl)=CC=C1)C(=O)OC
FENOFIBRATE	0	CC(C)OC(=O)C(C)(C)OC1=CC=C(C=C1)C(=O)C1=CC=C(Cl)C=C1
FENTANYL	1	CCC(=O)N(C1CCN(CCC2=CC=CC=C2)CC1)C1=CC=CC=C1
FEXOFENADINE	1	CC(C)(C(O)=O)C1=CC=C(C=C1)C(O)CCCN1CCC(CC1)C(O)(C1=CC=CC=C1)C1=CC=CC=C1
FLUCONAZOLE	0	OC(CN1C=NC=N1)(CN1C=NC=N1)C1=C(F)C=C(F)C=C1
FLUOROURACIL	0	FC1=CNC(=O)NC1=O
FLUOXETINE	0	CNCCC(OC1=CC=C(C=C1)C(F)(F)F)C1=CC=CC=C1
FOSINOPRIL	0	CCC(=O)O[C@@H](OP(=O)(CCCCC1=CC=CC=C1)CC(=O)N1C[C@@H](C[C@H]1C(O)=O)C1CCCCC1)C(C)C
FUROSEMIDE	1	NS(=O)(=O)C1=C(Cl)C=C(NCC2=CC=CO2)C(=C1)C(O)=O
GABAPENTIN	0	NCC1(CC(O)=O)CCCCC1
GLIMEPIRIDE	0	CCC1=C(C)CN(C(=O)NCCC2=CC=C(C=C2)S(=O)(=O)NC(=O)N[C@H]2CC[C@H](C)CC2)C1=O
GLYBURIDE	0	COC1=C(C=C(Cl)C=C1)C(=O)NCCC1=CC=C(C=C1)S(=O)(=O)NC(=O)NC1CCCCC1
HYDRALAZINE	0	NNC1=NN=CC2=CC=CC=C12
HYDROCHLOROTHIAZIDE	0	NS(=O)(=O)C1=C(Cl)C=C2NCNS(=O)(=O)C2=C1
HYDROCORTISONE	0	[H][C@@]12CC[C@](O)(C(=O)CO)[C@@]1(C)C[C@H](O)[C@@]1([H])[C@@]2([H])CCC2=CC(=O)CC[C@]12C
HYDROXYCHLOROQUINE	1	CCN(CCO)CCCC(C)NC1=C2C=CC(Cl)=CC2=NC=C1

IBRUTINIB	1	NC1=NC=NC2=C1C(=NN2[C@@H]1CCCN(C1)C(=O)C=C)C1=CC=C(OC2=CC=CC=C2)C=C1
IRBESARTAN	0	CCCCC1=NC2(CCCC2)C(=O)N1CC1=CC=C(C=C1)C1=CC=CC=C1C1=NNN=N1
IRINOTECAN	1	CCC1=C2CN3C(=CC4=C(COC(=O)[C@]4(O)CC)C3=O)C2=NC2=CC=C(OC(=O)N3CCC(CC3)N3CCCCC3)C=C12
ISONIAZID	0	NNC(=O)C1=CC=NC=C1
ISOSORBIDE	0	[H][C@]1(O)CO[C@]2([H])[C@]([H])(O)CO[C@]12[H]
ISOTRETINOIN	0	C\C(\C=C\C1=C(C)CCCC1(C)C)=C/C=C/C(/C)=C\C(O)=O
LABETALOL	0	CC(CCC1=CC=CC=C1)NCC(O)C1=CC(C(N)=O)=C(O)C=C1
LAMOTRIGINE	1	NC1=NC(N)=C(N=N1)C1=C(Cl)C(Cl)=CC=C1
LANSOPRAZOLE	1	CC1=C(OCC(F)(F)F)C=CN=C1CS(=O)C1=NC2=CC=CC=C2N1
LEUCOVORIN	1	[H]C(=O)N1C(CNC2=CC=C(C=C2)C(=O)N[C@@H](CCC(O)=O)C(O)=O)CNC2=C1C(=O)NC(N)=N2
LEVOFLOXACIN	1	C[C@H]1COC2=C3N1C=C(C(O)=O)C(=O)C3=CC(F)=C2N1CCN(C)CC1
LEVOTHYROXINE	0	N[C@@H](CC1=CC(I)=C(OC2=CC(I)=C(O)C(I)=C2)C(I)=C1)C(O)=O
LIDOCAINE	0	CCN(CC)CC(=O)NC1=C(C)C=CC=C1C
LINEZOLID	0	CC(=O)NC[C@H]1CN(C(=O)O1)C1=CC(F)=C(C=C1)N1CCOCC1
LOPERAMIDE	0	CN(C)C(=O)C(CCN1CCC(O)(CC1)C1=CC=C(Cl)C=C1)(C1=CC=CC=C1)C1=CC=CC=C1
LORATADINE	0	CCOC(=O)N1CCC(CC1)=C1C2=C(CCC3=C1N=CC=C3)C=C(Cl)C=C2
LORAZEPAM	1	OC1N=C(C2=CC=CC=C2Cl)C2=C(NC1=O)C=CC(Cl)=C2
LOSARTAN	1	CCCCC1=NC(Cl)=C(CO)N1CC1=CC=C(C=C1)C1=CC=CC=C1C1=NNN=N1
LOVASTATIN	0	[H][C@]12[C@H](C[C@@H](C)C=C1C=C[C@H](C)[C@@H]2CC[C@@H]1C[C@@H](O)CC(=O)O1)OC(=O)[C@@H](C)CC
MEFLOQUINE	0	OC(C1CCCCN1)C1=CC(=NC2=C1C=CC=C2C(F)(F)F)C(F)(F)F
MEROPENEM	0	[H][C@]1([C@@H](C)O)C(=O)N2C(C(O)=O)=C(S[C@@H]3CN[C@@H](C3)C(=O)N(C)C)[C@H](C)[C@]12[H]
METFORMIN	0	CN(C)C(=N)NC(N)=N
METHOTREXATE	0	[H][C@@](CCC(O)=O)(NC(=O)C1=CC=C(C=C1)N(C)CC1=NC2=C(N)NC(=N)N=C2N=C1)C(O)=O

METHYLPHENIDATE	0	COC(=O)C(C1CCCCN1)C1=CC=CC=C1
METOCLOPRAMIDE	0	CCN(CC)CCNC(=O)C1=CC(Cl)=C(N)C=C1OC
METOPROLOL	0	COCCC1=CC=C(OCC(O)CNC(C)C)C=C1
METRONIDAZOLE	1	CC1=NC=C(N1CCO)[N+]([O-])=O
MIDAZOLAM	0	CC1=NC=C2CN=C(C3=CC=CC=C3F)C3=C(C=CC(Cl)=C3)N12
MILRINONE	0	CC1=C(C=C(C#N)C(=O)N1)C1=CC=NC=C1
MINOCYCLINE	0	[H][C@@]12CC3=C(C(O)=CC=C3N(C)C)C(=O)C1=C(O)[C@]1(O)C(=O)C(C(N)=O)=C(O)[C@@H](N(C)C)[C@]1([H])C2
MINOXIDIL	0	NC1=CC(=NC(N)=[N+]1[O-])N1CCCCC1
MIRTAZAPINE	0	CN1CCN2C(C1)C1=CC=CC=C1CC1=C2N=CC=C1
MONTELUKAST	0	OC(=O)CC1(CC1)CS[C@H](CCC1=CC=CC=C1C(O)(C)C)C1=CC=CC(\C=C\C2=NC3=C(C=CC(Cl)=C3)C=C2)=C1
MOXIFLOXACIN	0	[H][C@]12CN(C[C@@]1([H])NCCC2)C1=C(F)C=C2C(=O)C(=CN(C3CC3)C2=C1OC)C(O)=O
MYCOPHENOLATE	0	COC1=C(C\C=C(/C)CCC(O)=O)C(O)=C2C(=O)OCC2=C1C
NAFCILLIN	1	[H][C@]12SC(C)(C)[C@@H](N1C(=O)[C@H]2NC(=O)C1=C(OCC)C=CC2=CC=CC=C12)C(O)=O
NAPROXEN	1	COC1=CC2=C(C=C1)C=C(C=C2)[C@H](C)C(O)=O
NIFEDIPINE	0	COC(=O)C1=C(C)NC(C)=C(C1C1=CC=CC=C1[N+]([O-])=O)C(=O)OC
NITROFURANTOIN	1	C1C(=O)NC(=O)N1/N=C/C2=CC=C(O2)[N+](=O)[O-]
NITROGLYCERIN	0	[O-][N+](=O)OCC(CO[N+]([O-])=O)O[N+]([O-])=O
NIZATIDINE	1	CNC(NCCSCC1=CSC(CN(C)C)=N1)=C[N+]([O-])=O
NOREPINEPHRINE	0	NC[C@H](O)C1=CC(O)=C(O)C=C1
NYSTATIN	0	C[C@H]1/C=C/C=C/CC/C=C/C=C/C=C/C=C/C(CC2C(C(C[C@](O2)(CC(C(CCC(CC(CC(CC(=O)O[C@H]([C@@H]([C@@H]1O)C)C)O)O)O)O)O)O)O)C(=O)O)O[C@@H]3[C@H]([C@H]([C@@H]([C@H](O3)C)O)N)O
OLANZAPINE	1	CN1CCN(CC1)C1=NC2=CC=CC=C2NC2=C1C=C(C)S2
OLMESARTAN	1	CCCC1=NC(=C(N1CC1=CC=C(C=C1)C1=C(C=CC=C1)C1=NN=NN1)C(O)=O)C(C)(C)O
OMEPRAZOLE	0	COC1=CC2=C(C=C1)N=C(N2)S(=O)CC1=NC=C(C)C(OC)=C1C

ONDANSETRON	1	CN1C2=C(C3=CC=CC=C13)C(=O)C(CN1C=CN=C1C)CC2
OXACILLIN	0	[H][C@]12SC(C)(C)[C@@H](N1C(=O)[C@H]2NC(=O)C1=C(C)ON=C1C1=CC=CC=C1)C(O)=O
OXALIPLATIN	1	C1CC[C@H]([C@@H](C1)[NH-])[NH-].C(=O)(C(=O)[O-])[O-].[Pt+4]
OXAPROZIN	1	OC(=O)CCC1=NC(=C(O1)C1=CC=CC=C1)C1=CC=CC=C1
OXYCODONE	0	COC1=C2O[C@H]3C(=O)CC[C@@]4(O)[C@H]5CC(C=C1)=C2[C@@]34CCN5C
PAPAVERINE	1	COC1=C(OC)C=C(CC2=NC=CC3=CC(OC)=C(OC)C=C23)C=C1
PAROXETINE	1	FC1=CC=C(C=C1)[C@@H]1CCNC[C@H]1COC1=CC2=C(OCO2)C=C1
PENICILLIN	0	[H][C@]12SC(C)(C)[C@@H](N1C(=O)[C@H]2NC(=O)CC1=CC=CC=C1)C(O)=O
PHENOBARBITAL	1	CCC1(C(=O)NC(=O)NC1=O)C1=CC=CC=C1
PIOGLITAZONE	0	CCC1=CN=C(CCOC2=CC=C(CC3SC(=O)NC3=O)C=C2)C=C1
PIPERACILLIN	1	[H][C@]12SC(C)(C)[C@@H](N1C(=O)[C@H]2NC(=O)[C@H](NC(=O)N1CCN(CC)C(=O)C1=O)C1=CC=CC=C1)C(O)=O
PREGABALIN	0	CC(C)C[C@H](CN)CC(O)=O
PROPRANOLOL	1	CC(C)NCC(O)COC1=CC=CC2=C1C=CC=C2
PROPYLTHIOURACIL	1	CCCC1=CC(=O)NC(=S)N1
QUETIAPINE	1	OCCOCCN1CCN(CC1)C1=NC2=CC=CC=C2SC2=CC=CC=C12
QUINAPRIL	0	CCOC(=O)[C@H](CCC1=CC=CC=C1)N[C@@H](C)C(=O)N1CC2=CC=CC=C2C[C@H]1C(O)=O
QUININE	1	[H][C@]1(C[C@@H]2CC[N@]1C[C@@H]2C=C)[C@H](O)C1=CC=NC2=CC=C(OC)C=C12
RABEPRAZOLE	0	COCCCOC1=C(C)C(CS(=O)C2=NC3=CC=CC=C3N2)=NC=C1
RALOXIFENE	0	OC1=CC=C(C=C1)C1=C(C(=O)C2=CC=C(OCCN3CCCCC3)C=C2)C2=C(S1)C=C(O)C=C2
RAMIPRIL	0	[H][C@@]12CCC[C@]1([H])N([C@@H](C2)C(O)=O)C(=O)[C@H](C)N[C@@H](CCC1=CC=CC=C1)C(=O)OCC
RANITIDINE	1	CNC(NCCSCC1=CC=C(CN(C)C)O1)=C[N+]([O-])=O
RIFABUTIN	0	CO[C@H]1\C=C\O[C@@]2(C)OC3=C(C2=O)C2=C(C(O)=C3C)C(=O)C(NC(=O)\C(C)=C/C=C/[C@H](C)[C@H](O)[C@@H](C)[C@@H](O)[C@@H](C)[C@H](OC(C)=O)[C@@H]1C)=C1NC3(CCN(CC3)CC(C)C)N=C21

RIFAMPIN	1	C[C@H]1/C=C/C=C(\C(=O)NC2=C(C(=C3C(=C2O)C(=C(C4=C3C(=O)[C@](O4)(O/C=C/[C@@H]([C@H]([C@H]([C@@H]([C@@H]([C@@H]([C@H]1O)C)O)C)OC(=O)C)C)OC)C)C)O)O)/C=N/N5CCN(CC5)C)/C
RIFAXIM	1	O=C1C2=C(O[C@@]1(O/C=C/[C@@H]([C@H]([C@H]([C@@H]([C@@H]([C@@H]([C@@H](O)[C@@H](C)/C=C/C=C(C)\C3=O)C)O)C)OC(C)=O)C)OC)C)C(C)=C(O)C4=C2C5=C(C(N3)=C4O)N6C(C=C(C)C=C6)=N5
RISPERIDONE	0	CC1=C(CCN2CCC(CC2)C2=NOC3=C2C=CC(F)=C3)C(=O)N2CCCCC2=N1
ROSUVASTATIN	0	CC(C)C1=NC(=NC(C2=CC=C(F)C=C2)=C1\C=C\[C@@H](O)C[C@@H](O)CC(=O)O)N(C)S(C)(=O)=O
SERTRALINE	1	CN[C@H]1CC[C@@H](C2=CC(Cl)=C(Cl)C=C2)C2=CC=CC=C12
SILDENAFIL	0	CCCC1=NN(C)C2=C1N=C(NC2=O)C1=CC(=CC=C1OCC)S(=O)(=O)N1CCN(C)CC1
SIMVASTATIN	1	[H][C@]12[C@H](C[C@@H](C)C=C1C=C[C@H](C)[C@@H]2CC[C@@H]1C[C@@H](O)CC(=O)O1)OC(=O)C(C)(C)CC
SOTALOL	0	CC(C)NCC(O)C1=CC=C(NS(C)(=O)=O)C=C1
SPIRONOLACTONE	1	[H][C@@]12CC[C@@]3(CCC(=O)O3)[C@@]1(C)CC[C@@]1([H])[C@@]2([H])[C@@]([H])(CC2=CC(=O)CC[C@]12C)SC(C)=O
SUCRALFATE	0	O.O[Al](O)O.O[Al](O)OS(=O)(=O)OC[C@H]1O[C@@](COS(=O)(=O)O[Al](O)O)(O[C@H]2O[C@H](COS(=O)(=O)O[Al](O)O)[C@@H](OS(=O)(=O)O[Al](O)O)[C@H](OS(=O)(=O)O[Al](O)O)[C@H]2OS(=O)(=O)O[Al](O)O)[C@@H](OS(=O)(=O)O[Al](O)O)[C@@H]1OS(=O)(=O)O[Al](O)O
SULFISOXAZOLE	1	CC1=NOC(NS(=O)(=O)C2=CC=C(N)C=C2)=C1C
SURAMIN	1	CC1=C(NC(=O)C2=CC(NC(=O)NC3=CC=CC(=C3)C(=O)NC3=C(C)C=CC(=C3)C(=O)NC3=C4C(C=C(C=C4S(O)(=O)=O)S(O)(=O)=O)=C(C=C3)S(O)(=O)=O)=CC=C2)C=C(C=C1)C(=O)NC1=C2C(C=C(C=C2S(O)(=O)=O)S(O)(=O)=O)=C(C=C1)S(O)(=O)=O
TACROLIMUS	1	CO[C@@H]1C[C@@H](CC[C@H]1O)\C=C(/C)[C@H]1OC(=O)[C@@H]2CCCCN2C(=O)C(=O)[C@]2(O)O[C@@H]([C@H](C[C@H]2C)OC)[C@H](C[C@@H](C)C\C(C)=C\[C@@H](CC=C)C(=O)C[C@H](O)[C@H]1C)OC
TAMOXIFEN	0	CC\C(=C(/C1=CC=CC=C1)C1=CC=C(OCCN(C)C)C=C1)C1=CC=CC=C1
TAMSULOSIN	0	CCOC1=CC=CC=C1OCCN[C@H](C)CC1=CC(=C(OC)C=C1)S(N)(=O)=O

TETRACYCLINE	0	[H][C@@]12C[C@@]3([H])C(=C(O)[C@]1(O)C(=O)C(C(N)=O)=C(O)[C@H]2N(C)C)C(=O)C1=C(O)C=CC=C1[C@@]3(C)O
TIROFIBAN	1	CCCCS(=O)(=O)N[C@@H](CC1=CC=C(OCCCCC2CCNCC2)C=C1)C(O)=O
TOBRAMYCIN	0	NC[C@H]1O[C@H](O[C@@H]2[C@@H](N)C[C@@H](N)[C@H](O[C@H]3O[C@H](CO)[C@@H](O)[C@H](N)[C@H]3O)[C@H]2O)[C@H](N)C[C@@H]1O
TOLTERODINE	0	CC(C)N(CC[C@H](C1=CC=CC=C1)C1=C(O)C=CC(C)=C1)C(C)C
TOPIRAMATE	0	[H][C@@]12CO[C@@]3(COS(N)(=O)=O)OC(C)(C)O[C@@]3([H])[C@]1([H])OC(C)(C)O2
TRAZODONE	1	ClC1=CC=CC(=C1)N1CCN(CCCN2N=C3C=CC=CN3C2=O)CC1
TRIAMTERENE	0	NC1=NC(N)=C2N=C(C(N)=NC2=N1)C1=CC=CC=C1
TRIAZOLAM	0	CC1=NN=C2CN=C(C3=CC=CC=C3Cl)C3=C(C=CC(Cl)=C3)N12
TRIMETHOPRIM	1	COC1=CC(CC2=CN=C(N)N=C2N)=CC(OC)=C1OC
URSODIOL	0	C[C@H](CCC(O)=O)[C@H]1CC[C@@]2([H])[C@]3([H])[C@@H](O)C[C@]4([H])C[C@H](O)CC[C@]4(C)[C@@]3([H])CC[C@]12C
VALACYCLOVIR	0	CC(C)[C@H](N)C(=O)OCCOCN1C=NC2=C1NC(N)=NC2=O
VALGANCICLOVIR	0	CC(C)[C@H](N)C(=O)OCC(CO)OCN1C=NC2=C1NC(N)=NC2=O
VALPROIC ACID	1	CCCC(CCC)C(O)=O
VERAPAMIL	1	COC1=C(OC)C=C(CCN(C)CCCC(C#N)(C(C)C)C2=CC(OC)=C(OC)C=C2)C=C1
VORICONAZOLE	1	C[C@@H](C1=NC=NC=C1F)[C@](O)(CN1C=NC=N1)C1=C(F)C=C(F)C=C1
WARFARIN	0	CC(=O)CC(C1=CC=CC=C1)C1=C(O)C2=C(OC1=O)C=CC=C2
ZOLMITRIPTAN	1	CN(C)CCC1=CNC2=CC=C(C[C@H]3COC(=O)N3)C=C12

External validation set

Name	Class	SMILES
AMIODARONE	1	CCCCC1=C(C(=O)C2=CC(I)=C(OCCN(CC)CC)C(I)=C2)C2=C(O1)C=CC=C2
ATENOLOL	1	CC(C)NCC(O)COC1=CC=C(CC(N)=O)C=C1
ATOVAQUONE	0	OC1=C([C@H]2CC[C@@H](CC2)C2=CC=C(Cl)C=C2)C(=O)C2=CC=CC=C2C1=O
AZITHROMYCIN	0	CC[C@H]1OC(=O)[C@H](C)[C@@H](O[C@H]2C[C@@](C)(OC)[C@@H](O)[C@H](C)O2)[C@H](C)[C@@H](O[C@@H]2O[C@H](C)C[C@@H]([C@H]2O)N(C)C)[C@](C)(O)C[C@@H](C)CN(C)[C@H](C)[C@@H](O)[C@]1(C)O

AZTREONAM	1	C[C@H]1[C@H](NC(=O)C(=N/OC(C)(C)C(=O)O)\C2=CSC([NH3+])=N2)C(=O)N1S([O-])(=O)=O
BENAZEPRIL	0	[H][C@@]1(CCC2=CC=CC=C2N(CC(O)=O)C1=O)N[C@@H](CCC1=CC=CC=C1)C(=O)OCC
BUDESONIDE	0	[H][C@@]12C[C@H]3OC(CCC)O[C@@]3(C(=O)CO)[C@@]1(C)C[C@H](O)[C@@]1([H])[C@@]2([H])CCC2=CC(=O)C=C[C@]12C
BUMETANIDE	1	CCCCNC1=C(OC2=CC=CC=C2)C(=CC(=C1)C(O)=O)S(N)(=O)=O
CEFAZOLIN	1	[H][C@]12SCC(CSC3=NN=C(C)S3)=C(N1C(=O)[C@H]2NC(=O)CN1C=NN=N1)C(O)=O
CEFTAZIDIME	1	[O-]C(=O)C1=C(CS[C@]2([H])[C@H](NC(=O)C(=N/OC(C)(C)C(O)=O)\C3=CSC(N)=N3)C(=O)N12)C[N+]1=CC=CC=C1
CILASTATIN	0	CC1(C)C[C@@H]1C(=O)N\C(=C/CCCCSC[C@H](N)C(O)=O)C(O)=O
CLONAZEPAM	0	[O-][N+](=O)C1=CC2=C(NC(=O)CN=C2C2=CC=CC=C2Cl)C=C1
DICLOFENAC	0	OC(=O)CC1=CC=CC=C1NC1=C(Cl)C=CC=C1Cl
FINASTERIDE	0	[H][C@@]12CC[C@H](C(=O)NC(C)(C)C)[C@@]1(C)CC[C@@]1([H])[C@@]2([H])CC[C@@]2([H])NC(=O)C=C[C@]12C
FLUVASTATIN	0	CC(C)N1C(\C=C\[C@H](O)C[C@H](O)CC(O)=O)=C(C2=CC=C(F)C=C2)C2=CC=CC=C12
GENTAMICIN	0	CNC(C)C1CCC(N)C(OC2C(N)CC(N)C(OC3OCC(C)(O)C(NC)C3O)C2O)O1
GLIPIZIDE	0	CC1=NC=C(N=C1)C(=O)NCCC1=CC=C(C=C1)S(=O)(=O)NC(=O)NC1CCCCC1
HALOPERIDOL	1	OC1(CCN(CCCC(=O)C2=CC=C(F)C=C2)CC1)C1=CC=C(Cl)C=C1
HYDROMORPHONE	0	[H][C@@]12OC3=C(O)C=CC4=C3[C@@]11CCN(C)[C@]([H])(C4)[C@]1([H])CCC2=O
IBUPROFEN	1	CC(C)CC1=CC=C(C=C1)C(C)C(O)=O
IPRATROPIUM	0	[H][C@]12CC[C@]([H])(C[C@@H](C1)OC(=O)C(CO)C1=CC=CC=C1)[N+]2(C)C(C)C
LEVETIRACETAM	0	CC[C@H](N1CCCC1=O)C(N)=O
LISINOPRIL	1	NCCCC[C@H](N[C@@H](CCC1=CC=CC=C1)C(O)=O)C(=O)N1CCC[C@H]1C(O)=O
LORACARBEF	1	N[C@@H](C(=O)N[C@H]1[C@H]2CCC(Cl)=C(N2C1=O)C(O)=O)C1=CC=CC=C1
MELOXICAM	0	CN1C(C(=O)NC2=NC=C(C)S2)=C(O)C2=C(C=CC=C2)S1(=O)=O
METHYLDOPA	0	C[C@](N)(CC1=CC=C(O)C(O)=C1)C(O)=O
METHYLPREDNISOLONE	1	[H][C@@]12CC[C@](O)(C(=O)CO)[C@@]1(C)C[C@H](O)[C@@]1([H])[C@@]2([H])C[C@H](C)C2=CC(=O)C=C[C@]12C

METOLAZONE	0	CC1NC2=CC(Cl)=C(C=C2C(=O)N1C1=CC=CC=C1C)S(N)(=O)=O
MORPHINE	0	[H][C@@]12OC3=C(O)C=CC4=C3[C@@]11CCN(C)[C@]([H])(C4)[C@]1([H])C=C[C@@H]2O
NEFAZODONE	0	CCC1=NN(CCCN2CCN(CC2)C2=CC(Cl)=CC=C2)C(=O)N1CCOC1=CC=CC=C1
OXCARBAZEPINE	1	NC(=O)N1C2=CC=CC=C2CC(=O)C2=C1C=CC=C2
PANTOPRAZOLE	1	COC1=C(OC)C(CS(=O)C2=NC3=C(N2)C=C(OC(F)F)C=C3)=NC=C1
PHENYTOIN	1	O=C1NC(=O)C(N1)(C1=CC=CC=C1)C1=CC=CC=C1
PRAVASTATIN	0	[H][C@]12[C@H](C[C@H](O)C=C1C=C[C@H](C)[C@@H]2CC[C@@H](O)C[C@@H](O)CC(O)=O)OC(=O)[C@@H](C)CC
PREDNISONE	0	[H][C@@]12CC[C@](O)(C(=O)CO)[C@@]1(C)CC(=O)[C@@]1([H])[C@@]2([H])CCC2=CC(=O)C=C[C@]12C
PROCAINAMIDE	1	CCN(CC)CCNC(=O)C1=CC=C(N)C=C1
PROPOXYPHENE	1	CCC(=O)O[C@@](CC1=CC=CC=C1)([C@H](C)CN(C)C)C1=CC=CC=C1
QUINIDINE	1	[H][C@@]12CCN(C[C@@H]1C=C)[C@]([H])(C2)[C@@H](O)C1=C2C=C(OC)C=CC2=NC=C1
RISEDRONIC ACID	0	OC(CC1=CN=CC=C1)(P(O)(O)=O)P(O)(O)=O
TERAZOSIN	0	COC1=C(OC)C=C2C(N)=NC(=NC2=C1)N1CCN(CC1)C(=O)C1CCCO1
TRAMADOL	0	COC1=CC=CC(=C1)[C@@]1(O)CCCC[C@@H]1CN(C)C
VALSARTAN	0	CCCCC(=O)N(CC1=CC=C(C=C1)C1=CC=CC=C1C1=NNN=N1)[C@@H](C(C)C)C(O)=O
VANCOMYCIN	1	CN[C@H](CC(C)C)C(=O)N[C@@H]1[C@H](O)C2=CC=C(OC3=C(O[C@@H]4O[C@H](CO)[C@@H](O)[C@H](O)[C@H]4O[C@H]4C[C@](C)(N)[C@H](O)[C@H](C)O4)C4=CC(=C3)[C@@H](NC(=O)[C@H](CC(N)=O)NC1=O)C(=O)N[C@@H]1C3=CC(=C(O)C=C3)C3=C(O)C=C(O)C=C3[C@H](NC(=O)[C@@H](NC1=O)[C@H](O)C1=CC(Cl)=C(O4)C=C1)C(O)=O)C(Cl)=C2
VENLAFAXINE	0	COC1=CC=C(C=C1)C(CN(C)C)C1(O)CCCCC1
ZOLPIDEM	0	CN(C)C(=O)CC1=C(N=C2C=CC(C)=CN12)C1=CC=C(C)C=C1

Table S2. Summary of the six types of molecular fingerprints used.

Fingerprints type	Abbreviation	Pattern type	Bit size
MACCS	MACCS	Structural features	166
Atom Pairs 2D	AP2D	Structural features	780
PubChem	PubChem	Structural features	881
Klekota-Roth	KRFP	Structural features	4860
CDK	CDK	Hash fingerprints	1024
CDK Extended	ExtFP	Hash fingerprints	1024

Table S3. Hyperparameters of the machine learning-based models.

Method	Hyperparameter	Value
SVM	penalty parameter *C*	{0.5, 0.6, 0.7, 0.8, 0.9, 1}
RF	number of estimators	{10, 50, 100}
	max features	{auto, sqrt, log2, 0.5}
k-NN	number of neighbors	{2–10}
ANN	hidden layer size	{10, 50, 100}
Adaboost	number of estimators	{10, 50, 100}
XGboost	maximum tree depth	{3, 5, 8, 10}
	minimum sum of instance weights in child	{1, 3, 5}

Table S4. Prediction results of the optimal classification models on training set by using the five-fold cross-validation.

Methods	Molecular features	SE	SP	ACC	MCC	AUC
SVM	RDKit+MACCS	0.537±0.034	0.646±0.026	0.580±0.013	0.185±0.028	0.591±0.014
	RDKit+CDK	0.550±0.023	0.649±0.023	0.590±0.014	0.205±0.026	0.599±0.012
	RDKit+PubChem	0.515±0.025	0.584±0.035	0.537±0.011	0.101±0.029	0.549±0.014
	RDKit+KRFP	0.690±0.027	0.508±0.025	0.590±0.016	0.205±0.029	0.599±0.014
	RDKit+AP2D	0.530±0.026	0.607±0.031	0.558±0.014	0.139±0.021	0.569±0.010
	RDKit+ExtFP	0.577±0.022	0.629±0.036	0.590±0.022	0.210±0.028	0.603±0.014
	10md+MACCS	0.571±0.020	0.512±0.033	0.527±0.019	0.085±0.037	0.541±0.018
	10md+CDK	0.539±0.035	0.659±0.024	0.587±0.014	0.206±0.026	0.599±0.0120
	10md+PubChem	0.564±0.032	0.575±0.032	0.559±0.014	0.141±0.021	0.569±0.011
	10md+KRFP	0.572±0.019	0.636±0.021	0.589±0.010	0.212±0.023	0.604±0.010
	10md+AP2D	0.571±0.045	0.533±0.042	0.532±0.021	0.110±0.038	0.552±0.019
	10md+ExtFP	0.579±0.028	0.629±0.036	0.588±0.018	0.215±0.030	0.604±0.015
	CC+MACCS	0.544±0.032	0.654±0.033	0.590±0.015	0.199±0.030	0.599±0.015
	CC+CDK	0.514±0.023	0.697±0.034	0.591±0.018	0.222±0.022	0.605±0.010
	CC+PubChem	0.561±0.030	0.596±0.032	0.568±0.020	0.162±0.030	0.578±0.014
	CC+KRFP	0.582±0.036	0.625±0.044	0.589±0.014	0.210±0.026	0.603±0.013
	CC+AP2D	0.440±0.017	0.692±0.019	0.555±0.020	0.137±0.034	0.566±0.016
	CC+ExtFP	0.550±0.029	0.664±0.026	0.602±0.017	0.218±0.029	0.607±0.015
k-NN	RDKit+MACCS	0.464±0.020	0.731±0.025	0.592±0.015	0.206±0.031	0.597±0.015
	RDKit+CDK	0.485±0.023	0.711±0.017	0.594±0.014	0.203±0.028	0.598±0.014
	RDKit+PubChem	0.690±0.023	0.566±0.021	0.627±0.010	0.261±0.022	0.628±0.011
	RDKit+KRFP	0.651±0.022	0.474±0.039	0.557±0.021	0.128±0.038	0.563±0.018
	RDKit+AP2D	0.634±0.044	0.539±0.031	0.585±0.019	0.175±0.040	0.586±0.019
	RDKit+ExtFP	0.487±0.021	0.692±0.009	0.583±0.017	0.183±0.027	0.590±0.012
	10md+MACCS	0.435± 0.023	0.749±0.029	0.584±0.011	0.195±0.025	0.592±0.011
	10md+CDK	0.481±0.015	0.718±0.031	0.596±0.008	0.207±0.021	0.600±0.010

	10md+PubChem	0.669±0.020	0.572±0.024	0.618±0.017	0.243±0.031	0.621±0.016
	10md+KRFP	0.646±0.026	0.509±0.044	0.574±0.013	0.158±0.028	0.578±0.015
	10md+AP2D	0.435±0.023	0.763±0.021	0.597±0.015	0.213±0.032	0.599±0.015
	10md+ExtFP	0.500±0.027	0.696±0.027	0.597±0.015	0.199±0.035	0.598±0.016
	CC+MACCS	0.626±0.032	0.571±0.028	0.599±0.016	0.198±0.037	0.598±0.019
	CC+CDK	0.481±0.027	0.706±0.027	0.592±0.018	0.192±0.041	0.593±0.020
	CC+PubChem	0.655±0.020	0.565±0.024	0.608±0.014	0.222±0.027	0.610±0.013
	CC+KRFP	0.466±0.021	0.737±0.011	0.598±0.008	0.215±0.021	0.601±0.009
	CC+AP2D	0.646±0.023	0.564±0.020	0.603±0.012	0.211±0.024	0.605±0.013
	CC+ExtFP	0.489±0.025	0.711±0.023	0.593±0.017	0.207±0.024	0.600±0.013
NB	RDKit+MACCS	0.506±0.022	0.610±0.047	0.558±0.020	0.117±0.039	0.558±0.019
	RDKit+CDK	0.520±0.016	0.624±0.024	0.566±0.014	0.145±0.026	0.572±0.013
	RDKit+PubChem	0.541±0.037	0.505±0.035	0.523±0.013	0.047±0.024	0.523±0.012
	RDKit+KRFP	0.604±0.030	0.567±0.026	0.571±0.021	0.174±0.027	0.585±0.014
	RDKit+AP2D	0.544±0.031	0.475±0.041	0.505±0.018	0.018±0.031	0.509±0.015
	RDKit+ExtFP	0.521±0.042	0.587±0.023	0.551±0.021	0.109±0.040	0.554±0.020
	10md+MACCS	0.477±0.023	0.565±0.030	0.519±0.013	0.042±0.022	0.521± 0.011
	10md+CDK	0.526± 0.028	0.592±0.020	0.558±0.008	0.118±0.018	0.559± 0.009
	10md+PubChem	0.559±0.033	0.493±0.024	0.524±0.017	0.053±0.031	0.526±0.015
	10md+KRFP	0.565±0.031	0.635±0.017	0.589±0.014	0.204±0.019	0.600±0.009
	10md+AP2D	0.613±0.030	0.421±0.035	0.499±0.015	0.037±0.036	0.517±0.017
	10md+ExtFP	0.487±0.021	0.593±0.024	0.537±0.018	0.081±0.025	0.540±0.012
	CC+MACCS	0.434±0.017	0.588±0.028	0.504±0.014	0.021±0.038	0.511±0.018
	CC+CDK	0.526±0.029	0.577±0.028	0.550±0.016	0.103±0.038	0.551±0.019
	CC+PubChem	0.558±0.032	0.525±0.020	0.535±0.020	0.083±0.020	0.541±0.010
	CC+KRFP	0.557±0.028	0.627±0.029	0.583±0.008	0.188±0.032	0.592±0.015
	CC+AP2D	0.494±0.018	0.593±0.014	0.537±0.008	0.088±0.017	0.543±0.008
	CC+ExtFP	0.493±0.030	0.596±0.026	0.545±0.011	0.090±0.030	0.545±0.014

AdaBoost	RDKit+MACCS	0.611±0.044	0.529±0.047	0.566±0.012	0.144±0.034	0.570±0.016
	RDKit+CDK	0.594±0.039	0.593±0.035	0.590±0.014	0.189±0.025	0.594±0.013
	RDKit+PubChem	0.539±0.042	0.614±0.046	0.570±0.009	0.156±0.020	0.576±0.010
	RDKit+KRFP	0.541±0.031	0.654±0.031	0.593±0.013	0.197±0.026	0.598±0.013
	RDKit+AP2D	0.572±0.030	0.576±0.024	0.568±0.015	0.148±0.023	0.574±0.010
	RDKit+ExtFP	0.557±0.031	0.619±0.031	0.580±0.015	0.180±0.029	0.588±0.014
	10md+MACCS	0.559±0.014	0.568±0.025	0.559±0.015	0.127±0.024	0.563±0.012
	10md+CDK	0.602±0.027	0.588±0.025	0.588±0.014	0.190±0.039	0.595±0.019
	10md+PubChem	0.586±0.027	0.558±0.028	0.566±0.011	0.145±0.019	0.572±0.009
	10md+KRFP	0.574±0.042	0.628±0.029	0.599±0.014	0.206±0.023	0.601±0.012
	10md+AP2D	0.601±0.033	0.601±0.043	0.594±0.008	0.205±0.025	0.601±0.013
	10md+ExtFP	0.624±0.031	0.566±0.027	0.587±0.019	0.193±0.041	0.595±0.021
	CC+MACCS	0.607±0.043	0.587±0.029	0.592±0.018	0.194±0.032	0.597±0.016
	CC+CDK	0.596±0.025	0.603±0.026	0.596±0.014	0.199±0.027	0.599±0.013
	CC+PubChem	0.615±0.024	0.558±0.034	0.577±0.015	0.173±0.039	0.587±0.020
	CC+KRFP	0.600±0.025	0.602±0.035	0.598±0.009	0.203±0.022	0.601±0.011
	CC+AP2D	0.580±0.046	0.572±0.046	0.570±0.010	0.152±0.023	0.576±0.011
	CC+ExtFP	0.604±0.029	0.587±0.023	0.589±0.009	0.192±0.020	0.595±0.009
RF	RDKit+MACCS	0.544±0.019	0.639±0.028	0.583±0.015	0.186±0.030	0.591±0.015
	RDKit+CDK	0.565±0.024	0.632±0.032	0.593±0.018	0.199±0.030	0.599±0.015
	RDKit+PubChem	0.530±0.019	0.635±0.032	0.576±0.021	0.168±0.043	0.583±0.020
	RDKit+KRFP	0.565±0.044	0.628±0.045	0.587±0.025	0.196±0.044	0.596±0.022
	RDKit+AP2D	0.565±0.025	0.613±0.029	0.579±0.018	0.180±0.018	0.589±0.017
	RDKit+ExtFP	0.536±0.043	0.648±0.031	0.583±0.015	0.188±0.031	0.592±0.015
	10md+MACCS	0.567±0.019	0.596±0.033	0.576±0.025	0.164±0.043	0.582±0.021
	10md+CDK	0.522±0.037	0.686±0.031	0.591±0.015	0.213±0.032	0.604±0.016
	10md+PubChem	0.537±0.035	0.604±0.035	0.563±0.017	0.143±0.038	0.570±0.019
	10md+KRFP	0.609±0.022	0.588±0.024	0.592±0.014	0.198±0.025	0.598±0.012
	10md+AP2D	0.585±0.038	0.618±0.044	0.594±0.013	0.203±0.023	0.601±0.011

	10md+ExtFP	0.545±0.029	0.656±0.036	0.590±0.018	0.204±0.028	0.600±0.013
	CC+MACCS	0.611±0.018	0.592±0.036	0.601±0.019	0.206±0.034	0.601±0.017
	CC+CDK	0.554±0.033	0.655±0.028	0.596±0.009	0.210±0.015	0.604±0.007
	CC+PubChem	0.650±0.019	0.557±0.028	0.598±0.015	0.210±0.032	0.603±0.016
	CC+KRFP	0.558±0.038	0.642±0.029	0.584±0.018	0.210±0.022	0.601±0.011
	CC+AP2D	0.599±0.032	0.602±0.030	0.597±0.011	0.204±0.025	0.601±0.013
	CC+ExtFP	0.585±0.029	0.621±0.046	0.594±0.021	0.208±0.040	0.603±0.020
XGBoost	RDKit+MACCS	0.586±0.022	0.603±0.044	0.590±0.023	0.189±0.038	0.595±0.019
	RDKit+CDK	0.588±0.017	0.609±0.028	0.593±0.017	0.199±0.036	0.598±0.018
	RDKit+PubChem	0.597±0.031	0.600±0.026	0.592±0.009	0.196±0.021	0.598±0.011
	RDKit+KRFP	0.606±0.036	0.574±0.037	0.588±0.013	0.181±0.026	0.590±0.013
	RDKit+AP2D	0.585±0.037	0.597±0.029	0.588±0.014	0.182±0.024	0.591±0.013
	RDKit+ExtFP	0.562±0.018	0.600±0.020	0.579±0.012	0.163±0.025	0.581±0.012
	10md+MACCS	0.562±0.021	0.628±0.021	0.587±0.020	0.192±0.034	0.595±0.017
	10md+CDK	0.602±0.034	0.605±0.038	0.598±0.021	0.206±0.045	0.603±0.022
	10md+PubChem	0.580±0.032	0.609±0.025	0.590±0.010	0.191±0.021	0.595±0.011
	10md+KRFP	0.593±0.036	0.600±0.037	0.592±0.020	0.193±0.031	0.596±0.014
	10md+AP2D	0.581±0.041	0.623±0.033	0.593±0.021	0.206±0.035	0.602±0.018
	10md+ExtFP	0.546±0.023	0.618±0.043	0.576±0.021	0.166±0.042	0.582±0.021
	CC+MACCS	0.642±0.027	0.583±0.021	0.607±0.008	0.226±0.023	0.613±0.011
	CC+CDK	0.594±0.024	0.609±0.024	0.603±0.014	0.204±0.027	0.602±0.013
	CC+PubChem	0.610±0.011	0.615±0.021	0.608±0.009	0.226±0.021	0.612±0.010
	CC+KRFP	0.619±0.027	0.614±0.033	0.611±0.012	0.233±0.025	0.617±0.010
	CC+AP2D	0.603±0.026	0.603±0.027	0.600±0.011	0.207±0.020	0.603±0.010
	CC+ExtFP	0.594±0.039	0.608±0.034	0.596±0.021	0.203±0.046	0.601±0.021
ANN	RDKit+MACCS	0.550±0.038	0.553±0.029	0.540±0.013	0.105±0.027	0.551±0.013
	RDKit+CDK	0.621±0.027	0.569±0.023	0.589±0.021	0.192±0.038	0.595±0.018
	RDKit+PubChem	0.577±0.037	0.518±0.024	0.545±0.008	0.096±0.020	0.548±0.010
	RDKit+KRFP	0.584±0.019	0.539±0.037	0.553±0.019	0.125±0.036	0.562±0.018

RDKit+AP2D	0.566±0.048	0.551±0.032	0.549±0.020	0.120±0.039	0.559±0.019
RDKit+ExtFP	0.654±0.028	0.539±0.017	0.591±0.022	0.195±0.038	0.597±0.019
10md+MACCS	0.557±0.045	0.477±0.046	0.506±0.032	0.035±0.029	0.517±0.014
10md+CDK	0.625±0.036	0.554±0.045	0.579±0.012	0.179±0.023	0.589±0.012
10md+PubChem	0.572±0.042	0.540±0.016	0.549±0.023	0.113±0.046	0.556±0.022
10md+KRFP	0.552±0.032	0.608±0.038	0.569±0.009	0.163±0.022	0.580±0.010
10md+AP2D	0.565±0.038	0.505±0.024	0.523±0.028	0.072±0.032	0.535±0.017
10md+ExtFP	0.622±0.045	0.567±0.048	0.589±0.011	0.190±0.026	0.595±0.013
CC+MACCS	0.546±0.026	0.541±0.040	0.533±0.022	0.087±0.043	0.543±0.021
CC+CDK	0.634±0.015	0.552±0.018	0.590±0.008	0.187±0.012	0.593±0.006
CC+PubChem	0.537±0.042	0.570±0.024	0.542±0.017	0.107±0.032	0.553±0.016
CC+KRFP	0.585±0.031	0.595±0.021	0.577±0.022	0.182±0.028	0.590±0.013
CC+AP2D	0.520±0.022	0.580±0.038	0.534±0.022	0.101±0.040	0.550±0.020
CC+ExtFP	0.633±0.031	0.551±0.042	0.585±0.020	0.185±0.034	0.592±0.017

Table S5. Prediction results of RDMD+PubChem-k-NN and CCMD+KPFP-XGBoost models for external validation dataset.

**Name**	**True label**	**Model prediction**	
		**RDMD+PubChem-k-NN**	**CCMD+KPFP-XGBoost**
AMIODARONE	1	0	0
ATENOLOL	1	1	0
ATOVAQUONE	0	0	0
AZITHROMYCIN	0	0	0
AZTREONAM	1	1	1
BENAZEPRIL	0	0	0
BUDESONIDE	0	1	1
BUMETANIDE	1	1	1
CEFAZOLIN	1	1	1
CEFTAZIDIME	1	1	1
CILASTATIN	0	0	0
CLONAZEPAM	0	0	0
DICLOFENAC	0	1	1
FINASTERIDE	0	0	0
FLUVASTATIN	0	0	0
GENTAMICIN	0	0	0
GLIPIZIDE	0	0	0
HALOPERIDOL	1	1	0
HYDROMORPHONE	0	0	0
IBUPROFEN	1	1	0
IPRATROPIUM	0	0	0
LEVETIRACETAM	0	1	0
LISINOPRIL	1	0	0
LORACARBEF	1	1	1
MELOXICAM	0	0	0
METHYLDOPA	0	0	0

METHYLPREDNISOLONE	1	1	1
METOLAZONE	0	0	1
MORPHINE	0	1	0
NEFAZODONE	0	1	0
OXCARBAZEPINE	1	1	1
PANTOPRAZOLE	1	1	0
PHENYTOIN	1	1	1
PRAVASTATIN	0	0	0
PREDNISONE	0	1	1
PROCAINAMIDE	1	1	1
PROPOXYPHENE	1	1	1
QUINIDINE	1	1	1
RISEDRONIC ACID	0	0	0
TERAZOSIN	0	1	0
TRAMADOL	0	1	0
VALSARTAN	0	0	0
VANCOMYCIN	1	0	1
VENLAFAXINE	0	0	0
ZOLPIDEM	0	0	0

Table S6. The comparison results of the PubChem fingerprint between Levetiracetam and its corresponding tautomeric form.

Structures / Bits	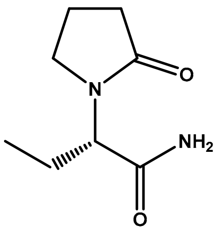	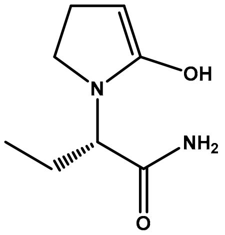 (Tautomeric form)
145	1	0
146	1	0
148	0	1
149	0	1
308	0	1
406	0	1
415	0	1
434	0	1
441	0	1
446	0	1
449	0	1
490	0	1
498	0	1
502	0	1
556	0	1
645	1	0
646	1	0
685	1	0

Table S7. The comparison results of the RDKit descriptors between Levetiracetam and its corresponding tautomeric form and protonation state.

Structures / Descriptors	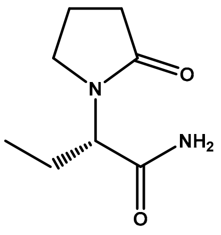	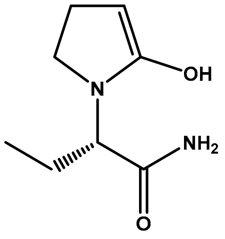 (Tautomeric form)
MaxEStateIndex	11.197	10.905
MinEStateIndex	-0.396	-0.376
MinAbsEStateIndex	0.056	0.181
qed	0.641	0.642
FpDensityMorgan1	1.750	1.917
FpDensityMorgan3	2.750	2.917
BalabanJ	2.647	2.664
BertzCT	203.235	213.103
Chi1n	4.253	4.115
Chi3v	2.201	2.083
Chi4n	1.574	1.455
HallKierAlpha	-1.060	-1.190
Kappa3	1.571	1.519
PEOE_VSA1	10.634	15.740
PEOE_VSA11	0.000	5.883
PEOE_VSA12	11.814	5.907
PEOE_VSA2	9.589	4.795
PEOE_VSA7	12.842	18.918
PEOE_VSA8	12.966	6.545
SMR_VSA1	9.589	9.901
SMR_VSA10	11.814	5.907
SMR_VSA5	32.228	25.807

SMR_VSA7	0.000	11.959
SlogP_VSA2	29.301	28.500
SlogP_VSA3	9.589	4.795
SlogP_VSA5	26.186	19.765
TPSA	63.400	66.560
EState_VSA1	5.907	0.000
EState_VSA2	11.949	17.832
EState_VSA3	19.386	12.966
EState_VSA6	4.900	10.976
VSA_EState9	32.667	32.167
FractionCSP3	0.750	0.625
NHOHCount	2	3
NumHAcceptors	2	3
NumSaturatedHeterocycles	1	0
NumSaturatedRings	1	0
MolLogP	-0.127	0.355
fr_C_O	2	1
fr_Ndealkylation2	1	0
fr_amide	2	1
**Structures** / **Descriptors**	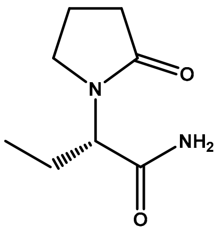	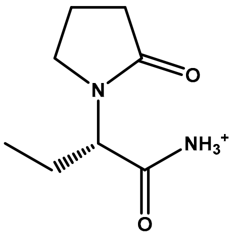 **(Protonation state)**
MaxEStateIndex	11.197	11.225
MinEStateIndex	-0.396	-0.278
MinAbsEStateIndex	0.056	0.096
qed	0.641	0.601
MolWt	170.212	171.220

Chi1n	4.253	4.317
Chi3v	2.201	2.245
Chi4n	1.574	1.620
HallKierAlpha	-1.060	-0.900
Kappa3	1.571	1.637
PEOE_VSA1	10.634	4.900
PEOE_VSA12	11.814	5.907
PEOE_VSA14	0.000	5.907
PEOE_VSA2	9.589	10.528
PEOE_VSA3	0.000	4.795
SMR_VSA1	9.589	15.323
SMR_VSA4	5.734	0.000
TPSA	63.400	65.020
EState_VSA1	5.907	0.000
EState_VSA2	11.949	17.856
EState_VSA3	19.386	12.842
EState_VSA4	6.421	12.966
EState_VSA8	0.000	5.734
EState_VSA9	5.734	0.000
VSA_EState9	32.667	31.667
NHOHCount	2	3
MolLogP	-0.127	-0.844
fr_priamide	1	0
fr_quatN	0	1
